# Genome-wide identification of the *ARF* (auxin response factor) gene family in peach and their expression analysis

**DOI:** 10.1007/s11033-020-05525-0

**Published:** 2020-05-19

**Authors:** Donghui Diao, Xiao Hu, Dan Guan, Wei Wang, Haiqing Yang, Yueping Liu

**Affiliations:** 1 College of Bioscience and Resources Environment, Beijing University of Agriculture, Beijing, 102206 China; 2 College of Plant Science and Technology, Beijing University of Agriculture, Beijing, 102206 China; 3 Pinggu District of Fruit Bureau, Beijing, 101200 China; 4 Key Laboratory for Northern Urban Agriculture Ministry of Agriculture and Rural Affairs, Beijing University of Agriculture, Beijing, 102206 China; 5 Beijing Advanced Innovation Center for Tree Breeding by Molecular Design, Beijing University of Agriculture, Beijing, 102206 China

**Keywords:** Peach, Auxin, *ARF* genes, Expression analysis

## Abstract

Auxin response factors (ARFs) are important transcription factors to relay auxin signaling. From the Genome Database for Rosaceae (GDR), we identified 17 peach *ARF* genes (*PpARF*s) encoding the proteins with three conserved domains. Their gene structure and functional domains were analyzed. Their transcriptional response to exogenous auxin treatment was tested and confirmed. We also expressed *PpARF-GFP* fusion reporters in tobacco leaves and observed their nuclear localization by fluorescence microscopy. It has been known that ARFs are widely involved in fruit development. We compared the expression pattern of all *PpARF*s in different tissues including the fruits at different developmental stages of two peach cultivars, “melting” and “stony hard”. We found eight *PpARFs* were more highly expressed in the “melting” peaches compared to “stony hard” peaches, while three *PpARFs* were more highly expressed in “stony hard” peaches. Among them, the expression difference of *PpARF4*, *PpARF7* and *PpARF12* was large, and their function in regulating fruit development and fruit quality was discussed. Our work provides a basis for further exploring the mechanisms underlying auxin regulated peach fruit ripening.

**Electronic supplementary material:**

The online version of this article (10.1007/s11033-020-05525-0) contains supplementary material, which is available to authorized users.

## Introduction

Auxin has long been recognized as a major regulator of various biological processes, including tropic growth, root architecture, phototropism, tissue and organ development [1–3], and fruit ripening [4–6]. Previous studies demonstrated that two types of transcription factor families are necessary for the auxin signal transduction pathway: the auxin response factor (ARF) family and the auxin/indole acetic acid (Aux/IAA) family [3]. ARF factors generally contain three domains. The N-terminal B3-like DNA-binding domain (DBD) is highly conserved and binds to auxin response elements (AuxREs) in the promoters of auxin-responsive genes [7, 8]. The middle region (MR) can activate or inhibit target genes, depending on its amino acid composition [7, 8] The C-terminal dimerization domain (CTD) contains motifs III and IV, which are also found in Aux/IAAs. At low auxin concentrations, these two motifs can mediate heterodimerization of ARFs and Aux/IAAs, which prevents the ARF factor from binding to AuxREs. At high auxin concentrations, Aux/IAA is degraded through the ubiquitination pathway and the ARF factor is released and binds to AuxREs of the promoter to activate or repress gene expression [7–9].

At present, the functions of some ARF factors have been studied in *Arabidopsis thaliana*, tomato (*Solanum lycopersicum*), apple (*Malus domestica*), papaya (*Carica papaya* L.), and other species [7, 10–16]. Studies have shown that *AtARF2/3/5/6/7/8/17/19* are involved in the regulation of plant morphological growth, such as apical bud formation, pollen wall synthesis, vascular bundle development, hypocotyl tropic movement, and adventitious root formation [17–21]. Among them, *AtARF2* and *AtARF19* are considered the key genes in auxin and ethylene signaling transduction pathway [19]. AtARF6 and AtARF8 regulate the expression of *JAZ/TAFY10A* which is controlled by jasmonic acid (JA) [19]. In addition, the *AtARF7* and *AtARF19* double-knockout mutant is severely impaired in lateral root formation. This phenotype can be recovered by over-expression of *LBD16*/*ASL18* (*lateral organ boundaries-domain16*/*asymmetric leaves2-like18*) and *LBD29*/*ASL16* in the double mutant, indicating that AtARF7 and AtARF19 regulate lateral root formation via direct activation of LBD/ASLs in *Arabidopsis* [21]. AtARF5 participates in the maintenance of apical meristem development by directly regulating *AtARR7* and *AtARR15*, and is involved in the development of leaf vascular bundle tissue by regulating *AtATHB8* [22, 23].

In tomato, ARFs play important roles in fruit development and maturation. To date, 21 ARFs have been identified [10], of these, *SlARF3/5/6/13/16/17* are highly expressed in the green and red fruit, and *SlARF1/2/4/7/8/11*/*14* are highly expressed during the fruit maturation period. They function as either negative or positive regulators. It is shown that inhibiting the expression of *SlARF8* leads to the development of larger tomato fruit, indicating a negative role of SlARF8 in fruit size control [24]. Similar results have been observed in *Arabidopsis* and eggplant [24, 25]. In addition, SlARF7 has been reported to be a negative regulator of early tomato fruit development [26]. SlARF4 indirectly controls sugar accumulation in tomato fruits by suppressing the activation of *SlGLK1* perhaps through binding the AuxREs in its promoter. The expression of *SlGLK1* induces large-scale synthesis of chlorophyll, so promotes fruit sugar accumulation [27]. On the contrary, SlARF10 enhances the expression of *SlGLK1* [28]. Some studies have shown that SlARF2A and SlARF2B are involved in regulating fruit softening process by controlling ethylene synthesis. The tomato mutant with reduced expression of *SlARF2* produces less climacteric ethylene while the key ripening regulators such as *RIN*, *CNR*, *NOR*, and *TAGL1* are dramatically down-regulated [29, 30]. In addition, down-regulation of *SlARF4* can slow the softening process [31].

Some progress has also been made in understanding the function of ARFs in the development of other fruits such as citrus, papaya, apple and plum. The genome of *Citrus sinensis* contains 19 ARFs and *CsARF8* and *CsARF12* are highly expressed in the late stage of fruit development [16]. Eleven *ARF* members were screened from *Carica papaya* L. and the expression of *CpARF1* was found to be significantly increased during fruit development [12]. A total of 29 ARF-encoding genes were also detected in *Malus* [13]. Among them, MdARF13 acts directly on the promoter of its target gene *MdDRF* and interacts with MdMYB10 to suppress anthocyanin synthesis in apples [32]. The peach (*Prunus persicae* L*.*) genome encodes 17 ARF-encoding genes, but their expression features are largely unknown except *PpARF11* (*ppa002230m*) and *PpARF5* (*ppa001179m*) [33].

Peach (*Prunus persica* L.) is a typical climacteric fruit, and fruit firmness is one of the most important traits. Based on its firmness, mature peaches are divided into three phenotypes “melting”, “non-melting”, and “stony hard” [34, 35]. The firmness is determined by the softening process/degree of the mesocarp. Previous research demonstrated that fruits of the non-softening, or stony hard, phenotype exhibit lower levels of auxin [36]. Therefore, given the central role of ARFs in auxin signal transduction, we hypothesize they may have a role in peach fruit softening regulation. Thus, we studied the structural features of the entire *ARF* gene family in peach and analyzed their expression patterns. Results from our research reveal the complexity of *PpARF* expression during the fruit ripening process.

## Materials and methods

### Plant materials

Experimental samples of the melting peach ‘Okubo’ and the hard peach ‘Jingyu’ were picked from the experimental orchard of Beijing University of Agriculture (Changping District, Beijing, China). The samples included roots, stems, new leaves, old leaves, flower buds, full-bloom flowers, and fruit at 37, 46, 55, 63, 70, 78, 84, 92, 98, and 110 days after full bloom (DAB). ‘Okubo’ was fully matured at 98 DAB, and ‘Jingyu’ was fully matured at 110 DAB. We divided the development and maturation of peach fruit into four periods [35]: the first rapid growth period (1 to 37 DAB, S1), the hard core stage (38 to 63 DAB, S2), the second rapid growth period (‘Okubo’, 64 to 84 DAB; ‘Jingyu’, 64 to 92 DAB, S3), and mature period (‘Okubo’, after 84 DAB; ‘Jingyu’, after 92 DAB, S4). The mature period was further divided into S4-1 (‘Okubo’, 84 to 92 DAB; ‘Jingyu’, 92 to 98 DAB), S4-2 (‘Okubo’, 92to 98 DAB; ‘Jingyu’, 98 to 110 DAB), and S4-3 (‘Okubo’, after 98 DAB; ‘Jingyu’, after 110 DAB).

Fruits with no obvious pests, disease, or mechanical damage were randomly harvested. Some samples were directly treated in the field, and the others were stored at − 80 °C until further analysis.

### Identification of *ARF* genes in peach

We acquired the CDS sequences and protein sequences of *Arabidopsis thaliana* [37] and *Solanum lycopersicum* (retrieved from NCBI) ARF family members. We used protein sequences of *Arabidopsis thaliana* to identify peach homologs via BLAST+ algorithms in the Genome Database for Rosaceae (GDR, https://www.rosaceae.org/species/prunus/all). They were named as *PpARF*s and genetic annotation was obtained directly from GDR.

We compared the amino acid sequences of peach and tomato *ARF* genes using MEGA 5.0 software (5.0; MEGA Inc., Englewood, NJ, USA) and phylogenetic reconstruction was conducted with the Neighbor-Joining (NJ) method, non-parametric bootstrapping was performed with a bootstrap replication value of 1000. Conserved domain analyses were performed using the Batch CD-Search tool from NCBI. The full length gene structures of *PpARF*s were determined using the GSDS2.0 web tool (https://gsds.cbi.pku.edu.cn/index.php). Multiple sequence alignments of PpARF*s* were performed using Clustalx 2 and figure images were generated using ESPript 3.0 (https://espript.ibcp.fr/ESPript/cgi-bin/ESPript.cgi). The conserved domains among all ARFs were analyzed using Weblogo (https://weblogo.berkeley.edu/logo.cgi). Prediction of nuclear localization signals was performed using ScanProsite (https://prosite.expasy.org/scanprosite/). The isoelectric points (pIs) and molecular weight (MWs) of PpARFs were estimated using ExPASy (https://www.expasy.org/).

### Quantitative real-time PCR analysis

Fresh samples were frozen in liquid nitrogen, and approximately 1 g of pre-cooled peach tissue was weighed and ground to powder for RNA extraction using a refrigerating mill (Retsch MM400). Total RNA was extracted using the Biomed EASYspin RNA Rapid Plant Kit and add DNase I (Beijing, China) for minimize the effect genomic DNA contamination. The first strand of cDNA was reverse transcribed using Takara’s RNase M-MLV kit (Beijing, China) according to the manufacturer’s instructions followed by quantitative real-time PCR analysis using the primers listed in Table 1. The primers for RT-qPCR analysis of the 17 *PpARFs* gene were designed using Primer 5.0 software.

**Table 1:** Primers for quantitative real-time PCR of *PpARF* genes family

Gene name	Peach gene ID	Forward primer (5′–3′)	Reverse primer (5′–3′)
*PpARF1*	*ppa002394m*	GCCGAGACATTTATCCATCACC	TGAGAAAGACTCCGTTACACCA
*PpARF2A*	*ppa001392m*	GCTCCGTGTTGGTGTTAGAC	GGCTTGTTCTTGGCTTGTAGTA
*PpARF2B*	*ppa022314m*	AGAAAATAATCAACCCGTGCCTC	GGCACTCATCAGCTTGTCGTT
*PpARF3*	*ppa002065m*	GTTCCAGCAAGCAATGAATCCAG	TGATGCTTGCTTTCCGCCAT
*PpARF4*	*ppa001557m*	CGGTGATGTGCTCTAATGCTACT	GCTACTCTCCTGCGGCTTATG
*PpARF5*	*ppa000946m*	GAAGGGCTGCTAAATGACCCAAGA	ATACAGCGAACACAACCAACGA
*PpARF6*	*ppa001179m*	TGCCTCTCAATCCCAGTCACC	TGAACCCATGAGACTGTGCAA
*PpARF7*	*ppa000708m*	AATTGAGCCTGTTGTAACTCC	TGCCAAAGTCATCTCCAAGCCAA
*PpARF8A*	*ppa001069m*	AGGCATCTTCTCACGACAGG	GATTTGCTCGCCGAATACCCA
*PpARF8B*	*ppa003267m*	CCTCAGCACCTTCTACAGCA	ATGGCACATTCATTTGTTGACT
*PpARF10A*	*ppa002082m*	CCAATCGGACGCTAACAAT	GTGCCTGAACTTCCATATCTC
*PpARF10B*	*ppa002195m*	GCAGCTCGTACTCTTTGGTC	TTTCTCTGCATTCCCATCCGA
*PpARF12*	*ppa002617m*	ACCTTTTGTGGCTTCTATACCTG	ATGGTTTTCACTTCTTTTGCCTT
*PpARF16*	*ppa002710m*	ATCAAGCATACCAGCCATCCA	GCTACAAACTGAAGGCATTGGA
*PpARF17*	*ppa003136m*	AACATTGGCAGTTCACAGTC	TGAAACGAACTTCTGCCAACC
*PpARF18*	*ppa002230m*	CAAAGCCAAGTAATACCCCGAT	TTTACACACTGGCTCGCTCT
*PpARF19*	*ppa000479m*	CAGCGAATGAGGACATACACC	TCGGTCCTCTAACTGTCCCT
*PpTEF-2*	*ppa001368m*	GTTGCCTTGGTCGGTCTTGA	ATTGAACAGCAACACGCACAA

The *translation elongation factor 2* (*PpTEF*2) gene was used as an internal reference while Takara SYBR Premix Ex Taq II (Beijing, China) along with the corresponding primers and cDNA were added into the reaction system for RT-qPCR [38]. A reaction containing no cDNA template was used as a negative control. All the reactions were run using an Applied Biosystems StepOne (48 well) instrument. The data were analyzed using -ΔΔCT method [39]. The expression levels of *PpARFs* from diverse samples were normalized with the expression of *PpTEF2*. The quantification of each cDNA was based on the comparative Ct method and was calculated as 2^−ΔΔCt^ [12]. GraphPad Prism 6.0 software was used to generate the figures.

### Hormonal treatments

Ten ‘Jingyu’ peach fruits at the S4-2 developmental stage were selected for hormonal treatments. The cylindrical mesocarp with a diameter of approximately 9 mm was extracted from three fruits using a puncher, and was cut into 2–3 mm thick discs with a scalpel. Approximately 10 g of discs were equilibrated in MS (Murashige and Skoog) liquid medium (pH 5.5) for 30 min and then placed in 0.5 Mm α-naphthylacetic acid (NAA) or 20 μM β-chlorophenoxyisobutyric acid (PCIB) solution for 1.5 h, 3 h, 6 h, and 12 h. Water was used as a control treatment. The treated discs were frozen in liquid nitrogen and stored at − 80 °C until further analysis.

### Subcellular localization of PpARF-GFP fusion proteins

Based on the results of the phylogeny analysis, we selected four genes (*PpARF4/6/10/12*) separately from the three large clades and two genes from the sub-clade of clade I for subcellular localization experiments. Peach cDNA was obtained using the same method as described for RT-qPCR, and was amplified by PCR using Takara LA Taq high fidelity DNA polymerase (Beijing, China). PCR primers (Table 2) were designed using Primer 5.0 software.

**Table 2:** Primers for subcellular localization of *PpARF4*/*6*/*10A*/*12*

Gene ID	Peach gene ID	Forward primer (5′–3′)	Reverse primer (5′–3′)
*PpARF4*	*ppa001557m*	AGAACACGGGGGACTCTAGAATGGAAATTGATCTGAACC	GACTGACCACCCGGGGATCCGACCCTGATTACTGTTGG
*PpARF6*	*ppa001179m*	AGAACACGGGGGACTCTAGAATGAGGCTCTCATCTGCTG	GACTGACCACCCGGGGATCCATACTCGAGTGACCCC
*PpARF10A*	*ppa002195m*	AGAACACGGGGGACTCTAGAATGGAGTACTCAGAGAGAAGC	GACTGACCACCCGGGGATCCAGCAAATATGCTCAAAGG
*PpARF12*	*ppa002617m*	AGAACACGGGGGACTCTAGAATGGCGAATCGAGAAGG	GACTGACCACCCGGGGATCCGTCCGAGCTTGTTACG

We constructed recombinant plasmids where *PpARF4/6/10A/12* cDNA was fused with *green fluorescent protein* (*GFP*) gene followed by cloning the fused *GFP* construct into pBI121 binary vector using the BM Seamless Cloning Kit from Biomed Company (Beijing, China). The resulting plasmid was transformed into *Agrobacterium tumefaciens* strain GV3101. The *Agrobacterium* infiltration solution was prepared and injected into tobacco (*Nicotiana benthamiana*) leaves. The plants were incubated in the dark for 12 h and then under normal day time cycle for 2–3 days. The expression of the fused GFP gene was driven by the CaMV 35S promoter in plants. The green fluorescent signal was observed by confocal microscopy (LEICATCS SP8, Germany).

## Results

### Genome-wide identification of *PpARF* genes in peach

In total, 17 *ARFs* were identified in the *Prunus persica* genome (Table 3). These genes were named according to their phylogenetic relationship with their homologues in tomato. Their coding sequences range from 1767 bp (*PpARF8B*) to 3420 bp (*PpARF19*). Thus, the size of the predicted PpARFs ranges from 588 to 1139 aa. Their MWs are within 73.469 to 126.163 kDa. Their predicted pIs range from 5.24 (PpARF5) to 7.59 (PpARF10A).

**Table 3:** *ARF* family genes in peach (*P. persica* L.)

Gene name	Genbank ID	Peach Gene ID	Location	Intron numbers	CDs length (bp)	Amino acid length	MW (KDA)	PI
*PPARF1*	XM_007225091.2	*ppa002394m*	NC_034009.1 (47497463–47506683)	13	2037	678	75.515	6.06
*PPARF2A*	XM_020563715.1	*ppa001392m*	NC_034013.1 (13246315–13251610)	13	2520	839	93.456	6.24
*PPARF2B*	XM_020570522.1	*ppa022314m*	NC_034016.1 (21641331–21647582)	11	2010	669	74.849	5.83
*PPARF3*	XM_007213730.2	*ppa002065m*	NC_034012.1 (2598668–2604558)	10	2169	722	78.848	6.35
*PPARF4*	XM_020565141.1	*ppa001557m*	NC_034014.1 (679512–6804558)	11	2412	803	88.793	5.96
*PPARF5*	XM_020559232.1	*ppa000946m*	NC_034009.1 (33696217–33702414)	14	2862	953	104.216	5.24
*PPARF6*	XM_020562547.1	*ppa001179m*	NC_034012.1 (4217260–4224122)	13	2664	887	98.498	6.15
*PPARF7*	XM_020567694.1	*ppa000708m*	NC_034015.1 (18452282–18459312)	13	3084	1027	118.305	6.62
*PPARF8A*	XM_007217628.2	*ppa001069m*	NC_034011.1 (19859395–19866776)	13	2760	919	101.273	5.95
*PPARF8B*	XM_020560148.1	*ppa003267m*	NC_034011.1 (729287..741224)	6	1767	588	93.959	5.86
*PPARF10A*	XM_020567087.1	*ppa002082m*	NC_034014.1 (7163727–7168010)	3	2160	719	79.306	7.59
*PPARF10B*	XM_007207991.2	*ppa002195m*	NC_034014.1 (23708250–23712299)	2	2124	707	78.815	6.23
*PPARF12*	XM_020564187.1	*ppa002617m*	NC_034013.1 (12202419–12207234)	14	1959	652	73.496	6.25
*PPARF16*	XM_020558694.1	*ppa002710m*	NC_034010.1 (24477046–24480781)	3	1926	641	73.693	6.69
*PPARF17*	XM_007225627.2	*ppa003136m*	NC_034009.1 (41847694–41852182)	1	1803	600	66.198	6.32
*PPARF18*	XM_020556641.1	*ppa002230m*	NC_034010.1 (22988125–22993218)	13	2097	698	77.559	6.47
*PPARF19*	XM_007225363.2	*ppa000479m*	NC_034009.1 (4658153–4665804)	13	3420	1139	126.163	6.22

### PpARFs group into three phylogenetic clades

**Fig. 1:**
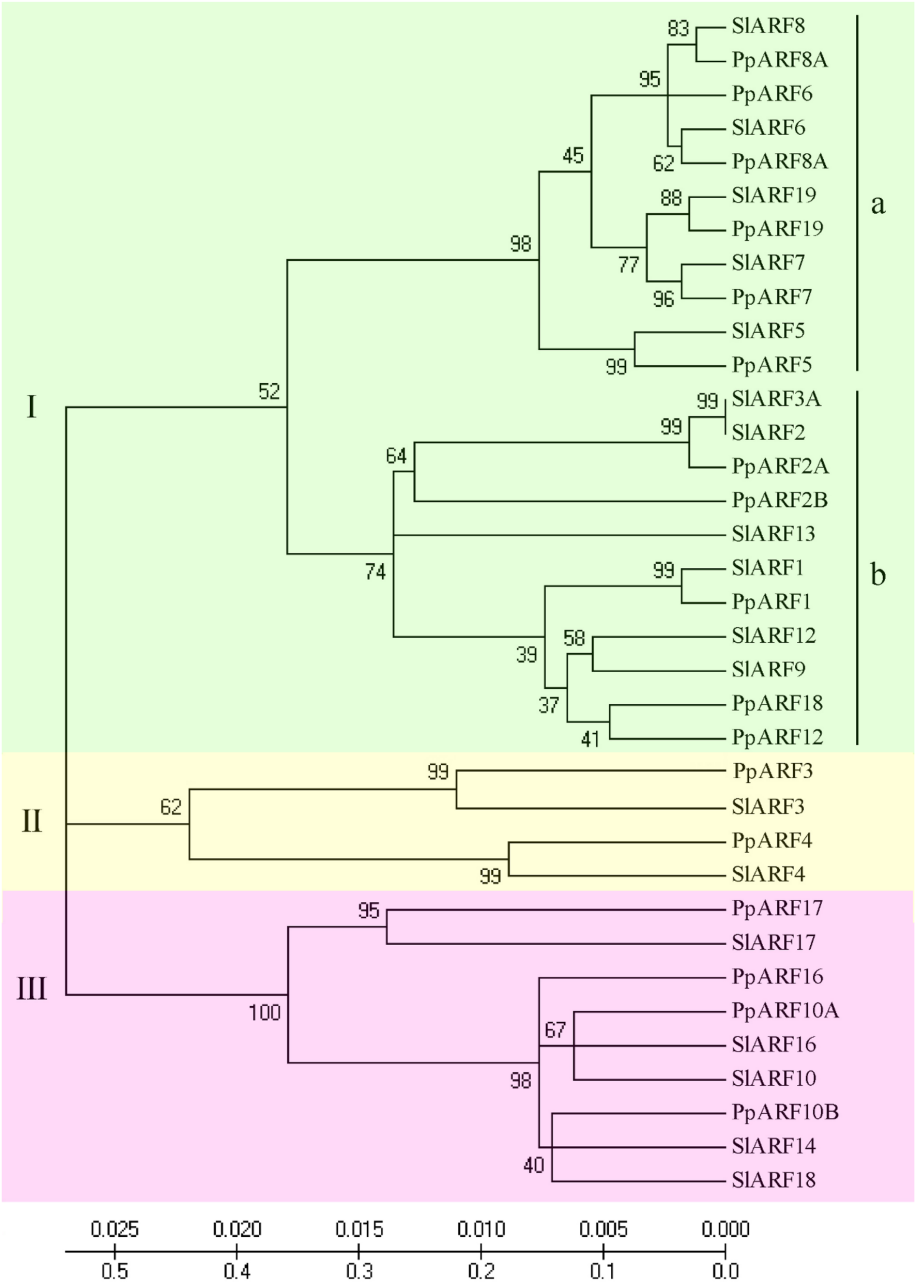
Phylogenetic relationship analysis. Phylogenetic tree of ARFs from peach (*P. persica* L.) and tomato (*S. lycopersicum*). ARFs were classified into three clades (I, II, and III). The tree was produced using MEGA 5.0 Bootstrap values from 1000 replicates are specified at each branch. The scale bar which represents the number of differences between sequences and the confidence from 0.000 to 0.025

The phylogenetic relationships among peach and tomato ARF proteins were investigated using cluster analysis. The tree shows that all 17 PpARFs grouped into three major clades (I, II, and III) (Fig. 1). Clade I includes two subclades: Ia and Ib (Fig. 1). Based on the phylogenetic tree, we identified 7 tomato-peach homolog pairs: *SlARF1/PpARF1*, *SlARF2/PpARF2A*, *SlARF3/PpARF3*, *SlARF4/PpARF4*,* SlARF5/PpARF5*, *SlARF7/PpARF7*, and *SlARF17/PpARF17*.

**Fig. 2:**
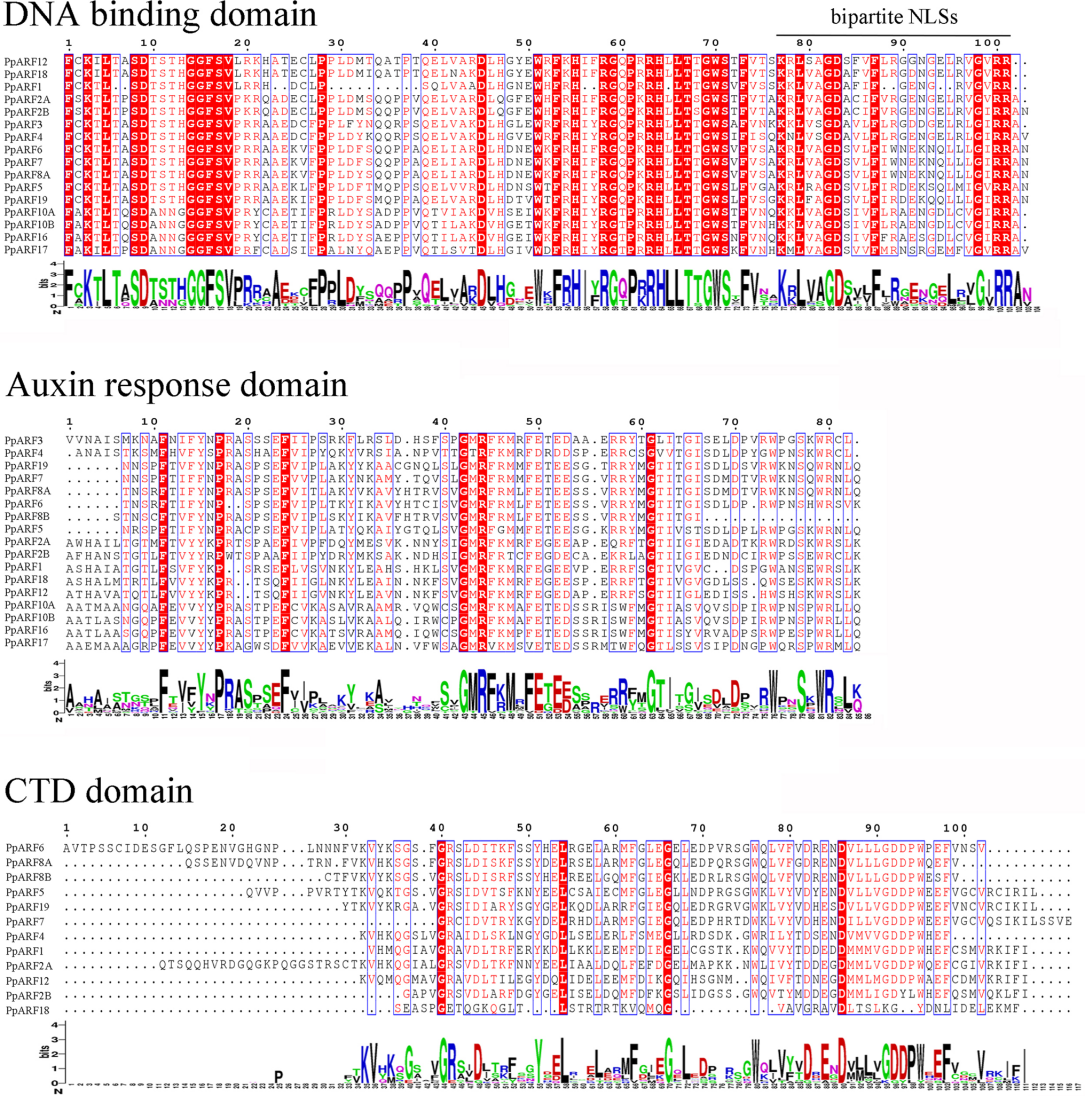
Conserved sequence analysis of PpARFs. The DNA-binding domain (16 PpARFs), auxin response domain (17 PpARFs), and CTD (12 PpARFs) are aligned. The ends of the DNA-binding domain include classical bipartite nuclear localization signals (NLSs)

The Multiple Expectation Maximization for Motif Elicitation (MEME) web server (https://meme-suite.org/index.html) was used to analyze the domain distributions in PpARFs. Three highly conserved domains (the DNA-binding domain, auxin response domain, and CTD) were identified. However, not all the PpARFs contain a CTD, such as PpARF3, PpARF10A, PpARF10B, PpARF16, and PpARF17 while the DNA-binding domain was not found in PpARF8A (Supplementary Fig. 1, Fig. 2). The gene structure of each *PpARF* was investigated by comparing the full-length CDS sequences with the corresponding genomic DNA sequences. The number of introns in *PpARF* genes ranged from 1 to 14 (Table 1, Online Resource 2).

### Representative PpARFs localize to the nucleus

**Fig. 3:**
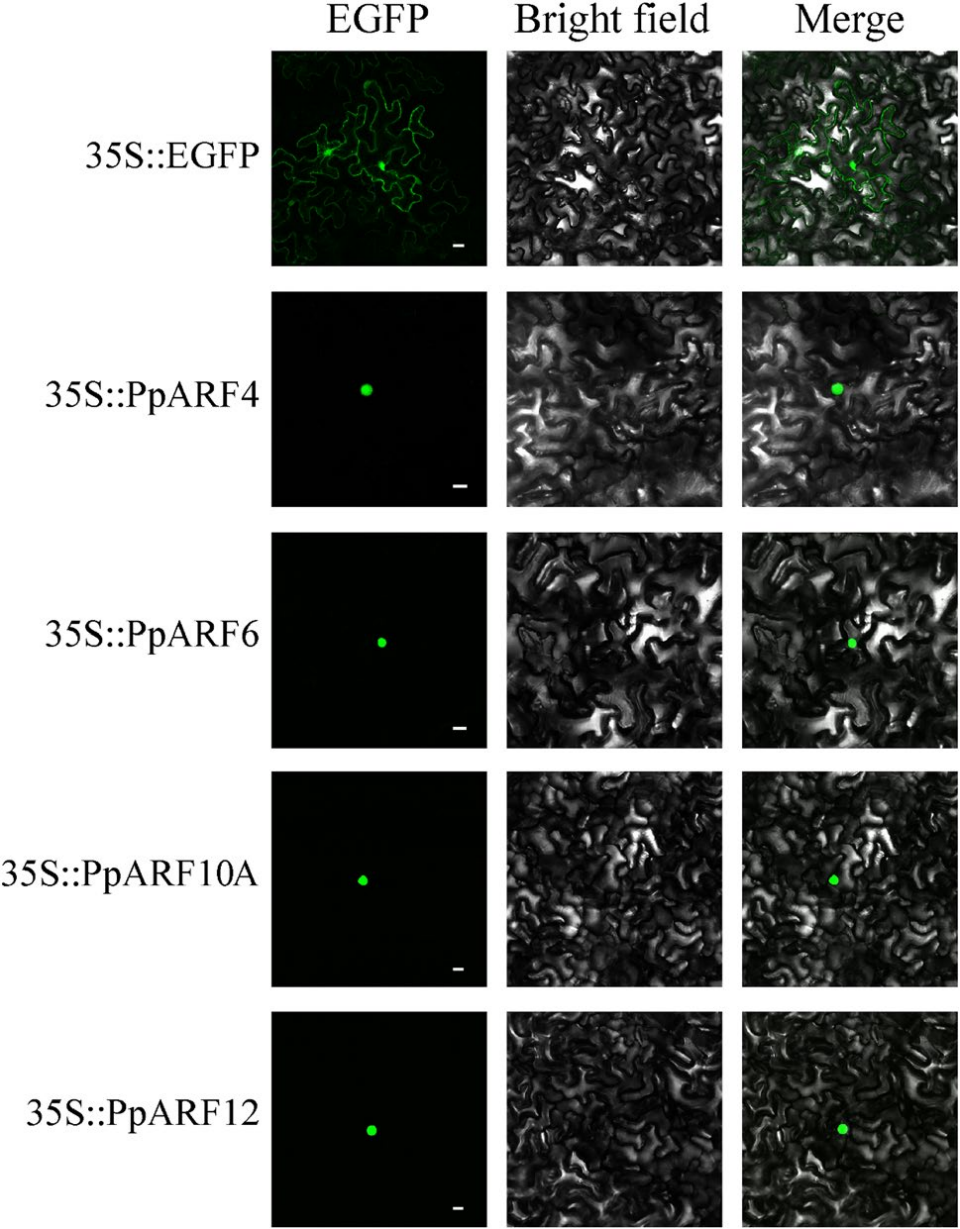
Subcellular localization of selected PpARFs. PpARFs-EGFP fusion proteins were transiently expressed in tobacco leaves and their cell nuclear localization was determined by confocal microscopy. The green fluorescent dot is the nucleus and the scale bar in each panel indicates 15.0 μM

Results from the analysis using the program Scan Prosite revealed that all PpARFs contain predicted bipartite nuclear localization signals (NLSs) [(K/R)(K/R)X10-12(K/R)3/5]. To confirm it, four PpARFs, PpARF4, PpARF6, PpARF10A and PpARF12, which represent the four different PpARF clades were selected for subcellular localization study. The 35S::PpARF-EGFP dual-expression vectors were constructed for each PpARF, and a 35S::EGFP construct was used as a positive control. Fluorescence microscopy revealed that the GFP signals of PpARF4, PpARF6, PpARF10A, and PpARF12 fusion proteins were only observed in the nucleus (Fig. 3) confirming that the selected PpARF proteins are nucleoproteins.

### *PpARF* genes are differentially expressed in specific tissues of peach

**Fig. 4:**
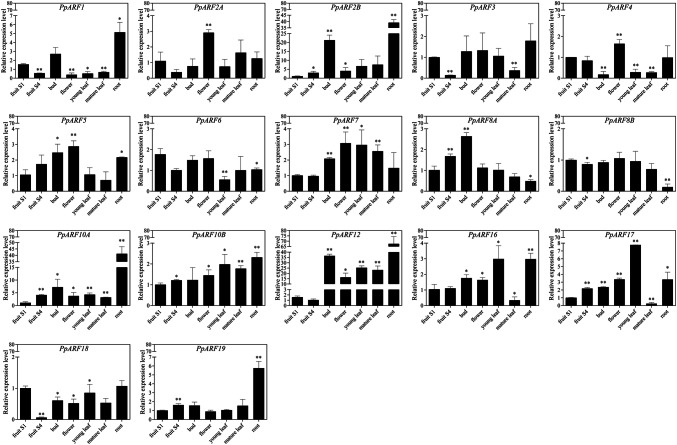
RT-qPCR analysis of *PpARFs* in different peach tissues and fruits at different developmental stages. The relative mRNA levels in S1 fruit were used as a reference (relative mRNA level was set as 1). All genes were analyzed using an algorithm based on the variances calculated by the cross-gene error model (± SD) in GraphPad Prism 6.0. Asterisks indicate statistically significant differences as determined by a Student’s *t*-test (*P < 0.05, **P < 0.01)

To determine the roles of *PpARFs* in peach growth and development, the expression of the 17 *PpARFs* were compared in different tissues and organs of the 'Okubo' melting cultivar, including the roots, young leaves, mature leaves, buds, flowers, and fruits in the S1 and S4-3 stages (Fig. 4). *PpARFs* were found to be ubiquitously expressed in all samples.

In peach fruit, the expression of *PpARF1*, *PpARF3*, and *PpARF18* was > fivefold reduced in S4 fruit compared to S1 fruit. In particular, *PpARF18* expression was decreased by > 15-fold. On the contrary, expression levels of *PpARF2B*, *PpARF8*, *PpARF17* and *PpARF19* were approximately twofold higher in S4-3 fruit than in S1 fruit while *PpARF10A* expression was about four times increased (Fig. 4). Hence, *PpARF18* and *PpARF10A* genes may both play more important roles in fruit ripening.

In other tissues or organs, the expression of *PpARF1* and *PpARF2B* showed an approximately 6–7fold increase and *PpARF12* expression was > twofold increased in buds compared to flowers. In the meantime, *PpARF2A* and *PpARF4* exhibited higher expression in flowers than in buds. In mature leaves, the expression of *PpARF16* and *PpARF17* was respectively decreased > 10fold and 30fold when compared to that in young leaves. In roots, the expression of *PpARF2B*, *PpARF10A*, and *PpARF12* were increased > 40-fold when compared to S1 fruit.

### Expression of *PpARFs* at different fruit development stages of two cultivars

**Fig. 5:**
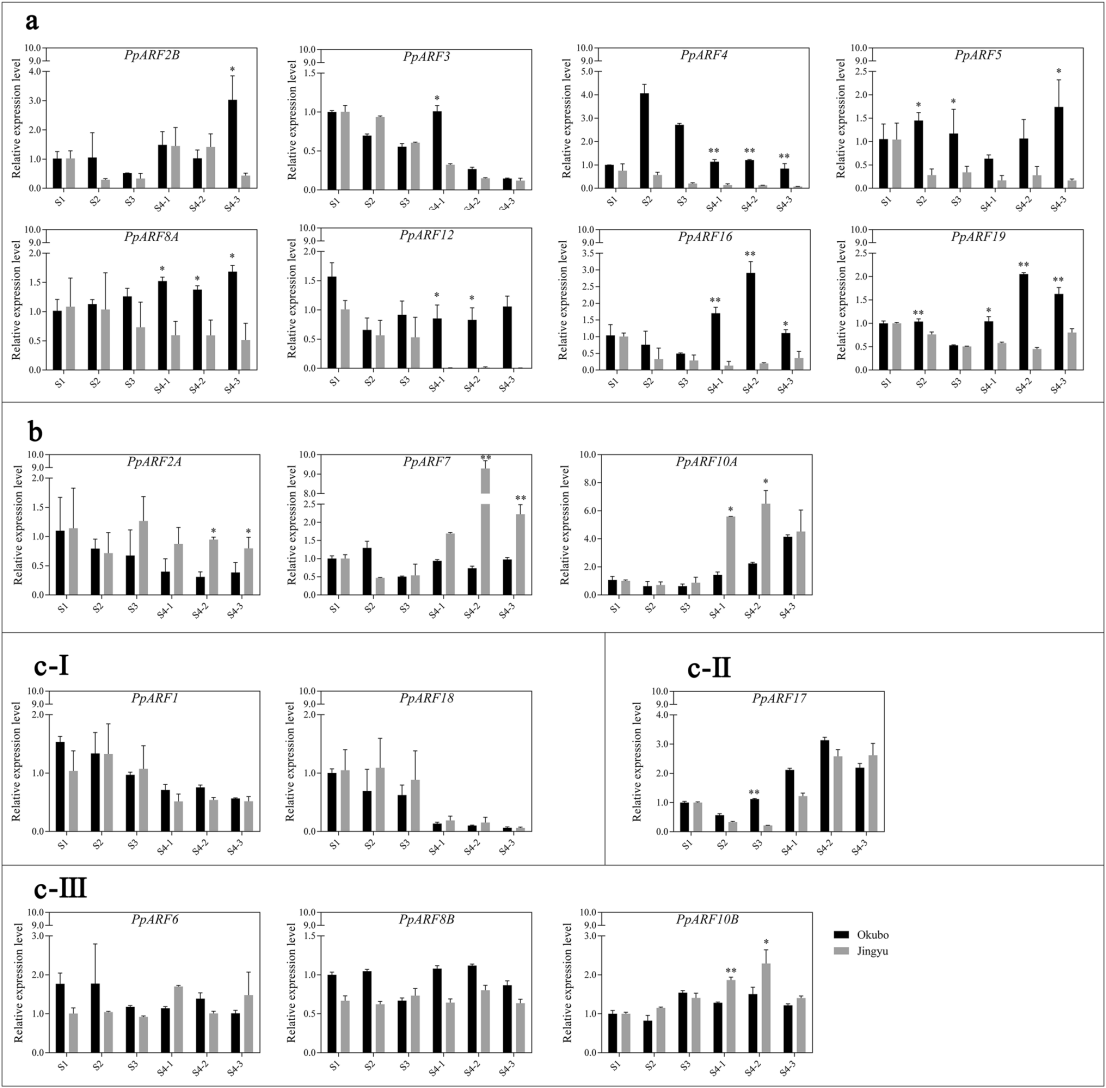
RT-qPCR analysis of *PpARF*s at different stages of fruit development in 'Okubo' (melting) and 'Jingyu' (stony hard) cultivars. **a** The *PpARFs* that were more highly expressed in 'Okubo' than in 'Jingyu' at maturity stages (S4-1 to S4-3). **b** The *PpARFs* that were more highly expressed in 'Jingyu' than in 'Okubo' at maturity stages (S4-1 to S4-3). **c** The *PpARFs* that displayed no significant difference between 'Okubo' and 'Jingyu'. The relative mRNA levels in 'Okubo' S1fruit were used as a reference (relative mRNA level set as 1). All genes were analyzed using an algorithm based on variances calculated by the cross-gene error model (± SD) in GraphPad Prism 6.0. Asterisks indicate statistically significant differences as determined by a Student’s *t*-test (*P < 0.05, **P < 0.01)

In order to figure out the effects of PpARFs on fruit maturation in ‘Okubo’ (melting) and ‘Jingyu’ (stony hard) cultivars, the expression levels of 17 *PpARFs* were analyzed by RT-qPCR. According to the expression differences at the S4 stage of the two varieties, the genes could be divided into three groups (Fig. 5), and group c could be further divided into three subgroups.Group a consists of 8 *PpARFs* whose expression levels were > twofold higher in ‘Okubo’ than ‘Jingyu’ at the S4 stage. Differences in *PpARF3* expression between two varieties were only observed at the S4-1 stage, and for *PpARF2B* and *PpARF5*, at the S4-3 stage. Differences in *PpARF4*, *PpARF8*, *PpARF12*, *PpARF16*, *PpARF19* expression were observed throughout the entire S4 stage (Fig. 5a). In particular, *PpARF12* expression was > 80-fold higher in ‘Okubo’ than in ‘Jingyu’. Group b includes three *PpARF*s. Their expression was higher in ‘Jingyu’ than in ‘Okubo’ at S4 stage (Fig. 5b). The expression of *PpARF10A* in ‘Jingyu’ was fivefold and threefold higher than that of ‘Okubo’ in S4-1and S4-2, respectively. The expression of *PpARF2A* and *PpARF7* in ‘Jingyu’ was higher than ‘Okubo’ at S4-2 and S4-3, respectively with *PpARF7* showing 12-fold higher expression. The rest of *PpARF*s belong to group c. We observed no significant differences in gene expression between the two varieties during the S4 stage. These genes were further divided into three subgroups according to their expression trends throughout fruit development. The gene expression of *PpARF*s in Group c-I gradually declined from S1 to S4-3, while Group c-II genes showed a gradual increase in expression from S1 to S4-3. Group c-III did not display any obvious changes in expression during fruit development and maturation.

**Fig. 6:**
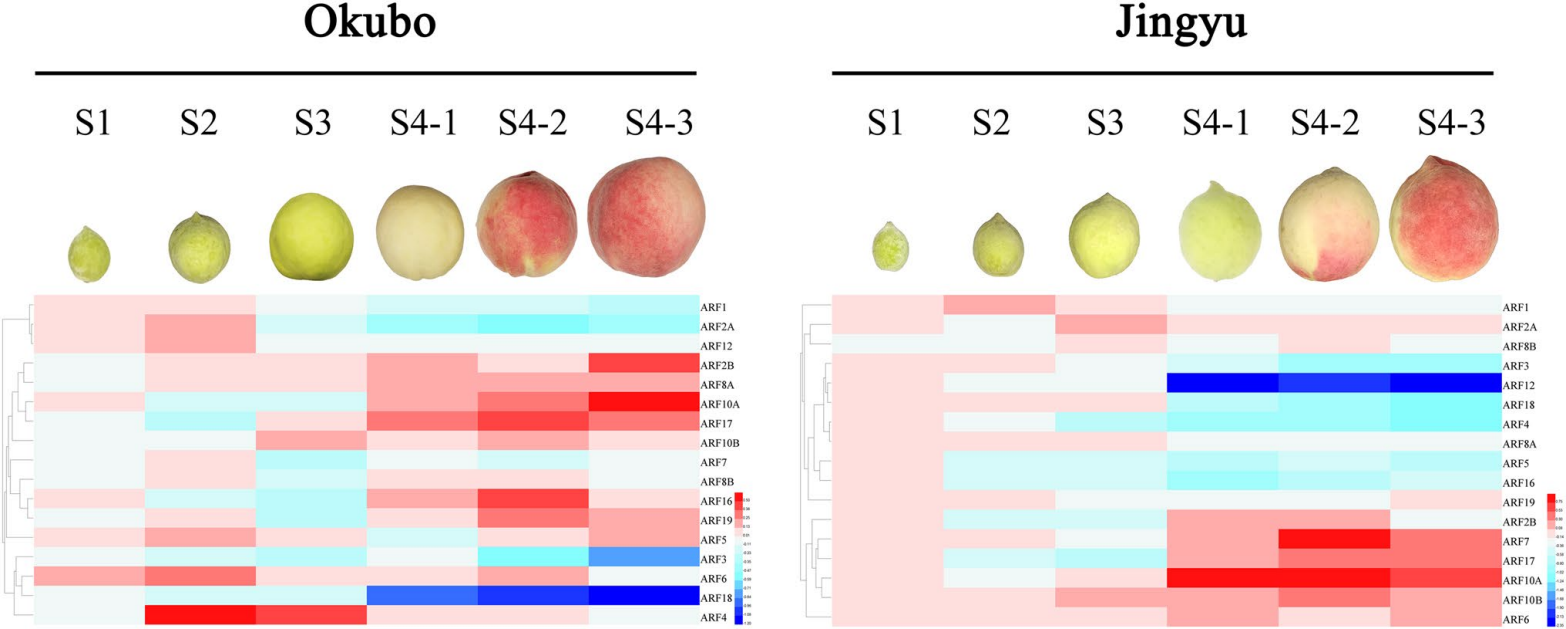
Heatmap of *PpARFs* expression in peach during different developmental stages in 'Okubo' and 'Jingyu' cultivars. Changes in the expression levels of *PpARF*s during different fruit developmental stages are relative to RNA accumulation levels. Levels of reduced (blue) or increased expression (red) are shown as average log values. The heatmap colors range from red to blue represent the expression level from the highest to the lowest, respectively. The images are generated by program of HemI. (Color figure online)

Our heatmap analysis (Fig. 6) more intuitively shows the dynamic changes of *ARF* gene expression at different stages of peach fruit development. For example, the expression of *PpARF12* in ‘Okubo’ was relatively stable in all stage, only slightly decreased in mature stage, However, in ‘Jingyu’, *PpARF12* expression decreased sharply at the mature stage. The expression pattern was opposite for *PpARF7* with reduced expression in 'Okubo' and increased expression in ‘Okubo’. The maximum difference in its expression between the two cultivars was observed at the S4-2 stage.

Given the above results, we think that the genes in Group a and b are important for peach fruit development and may be related to the softening trait of peach, particularly PpARF7 and PpARF12.

### Effects of in vitro hormone treatment on the expression of *PpARFs* in hard fruit

**Fig. 7:**
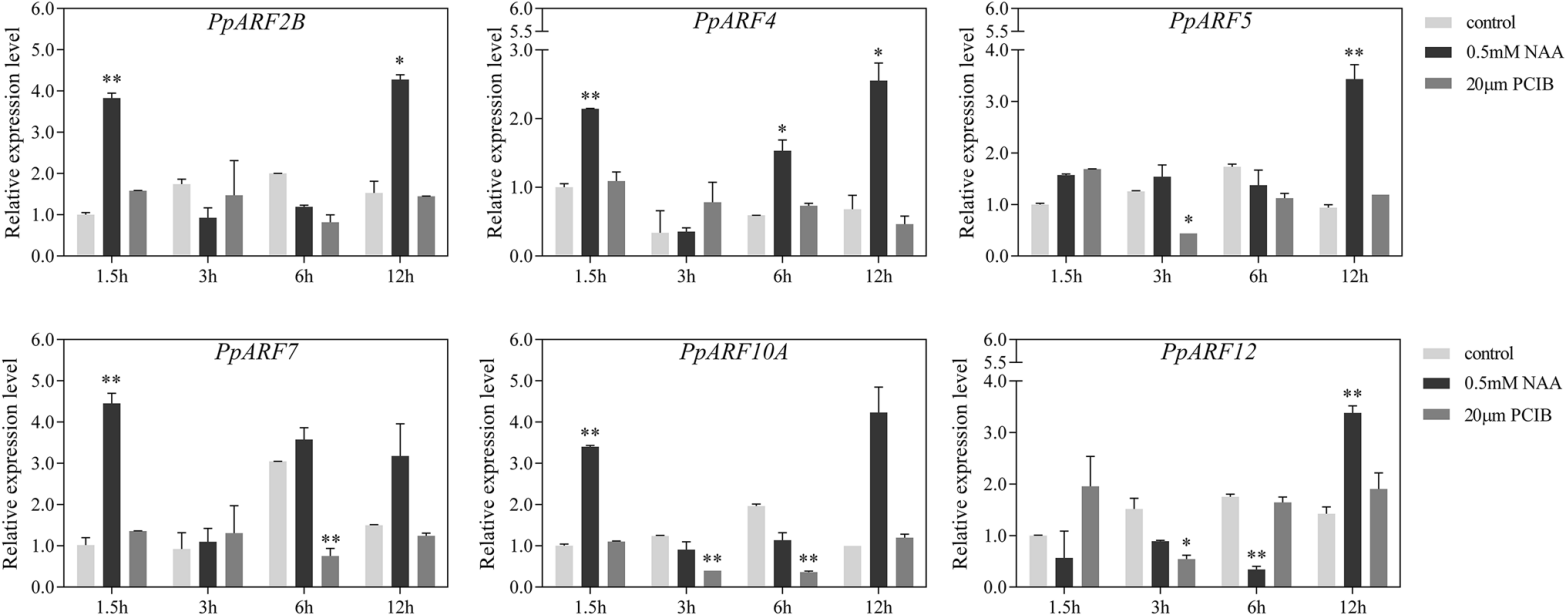
Expression of selected *PpARFs* at S4-2 stage of 'Jingyu' fruit under NAA or PCIB treatment. The relative mRNA levels of the control group at 1.5 h were used as a reference (relative mRNA level set as 1). All genes were analyzed using an algorithm based on variances calculated by the cross-gene error model (± SD) in GraphPad Prism 6.0. Asterisks indicate statistically significant differences as determined by a Student’s *t*-test (*P < 0.05, **P < 0.01)

ARFs are important factors in the auxin signal transduction pathway and are sensitive to auxin application. To investigate the effect of external application of auxin on the ARF family in peach, we treated ‘Jingyu’ peach fruit (mature S4-2 stage) in vitro with NAA and the auxin signal transduction inhibitor, PCIB to explore the effect of exogenous hormones on the transcription of *PpARFs* in the mesocarp of hard peach. The RT-qPCR results showed that the expression levels of *PpARF*s were significantly affected by exogenous NAA and PCIB (Fig. 7).

After 1.5 h of NAA treatment, the expression levels of *PpARF2B*, *PpARF4*, *PpARF7* and *PpARF10A* were more than doubled than those of the control group. These genes responded sensitively to auxin, but after 3 h of NAA exposure, their expression decreased to a level like the control group. By 6 h, only the expression of *PpARF4* was higher in the treated samples. However, *PpARF2B*, *PpARF4*, *PpARF7*, and *PpARF10A* expression was increased again after 12 h of NAA treatment. *PpARF5* and *PpARF12* displayed slow responses to NAA treatment up to12 h.

## Discussion

The ARF family is a key transcription factor family in plants [27]. Some previous work on *SlARF*s have indicated their regulatory roles in tomato fruit development. Thus, identification of the members in ARF family that have roles in regulating fruit development is important for other fruit crops or trees. In this study, we identified 17 *ARF*s in peach genome and characterized the features of their gene structure and conserved functional domains. Using real-time PCR analysis, we found some *PpARF* members that are specifically correlated with fruit development and maturation.

We have identified 17 ARF peach genes by blast search, however, in the latest research, 27 ARF peach genes were retrieved in genebank by *geneHummus*, which is a novel R package that efficiently and quickly identifies members of a plant gene family by searching for conserved domains [40]. The following 10 ARF peach protein (XM_020558695, XM_020566384, XM_020566385, XM_020566383, XM_007204881, XM_020567695, XM_020570523, XM_020559236, XM_007210839 and XM_020559243) were not identified in our study [33]. Interestingly, these 27 genes came from 17 loci, the mRNA sequences from the same locus are completely identical in some segments, and it is speculated that these genes of we have not identified are simply alternate gene models/splice-variants. Based on this, we compared the protein sequences from the same locus and found that some protein sequences are completely identical, such as ARF2A and XM_007210839, others show deletions or insertions of several or dozens of amino acids, such as ARF4 and XM_007204881. Splice-variant leads to polymorphisms in structure and function of transcripts and proteins, therefore, it would be interesting to check the expression and biological significance of the other 10 *ARF*s in our subsequent research.

A classical ARF factor contains three conserved domains: the DNA-binding domain, the auxin response domain, and the CTD. Among the total 17 ARF members, *PpARF8B* does not have a DNA-binding domain, indicating the possibility of losing the function as transcription factors. The auxin response domain was present in all PpARF proteins. Previous studies showed that glutamine-rich auxin response domains may have an activating function, and those rich in proline, threonine, and serine may have inhibitory functions [22, 41]. Twelve PpARFs (PpARF1/2A/2B/4/5/6/7/8A/8B/12/18/19) contain CTDs that bind to Aux/IAA, indicating the possible involvement of Aux/IAA in modulating their functions. Being the functional transcription factors, ARFs are localized in nucleus mainly through nuclear localization signal (NLS). Nevertheless, it is possible that functional transcriptional factors may be transported into nucleus by other cofactors. We investigated the NLS of four PpARFs, where two types of NLSs were found, and their nuclear localization was further confirmed. However, no NLS has been found in PpARF8B. Its subcellular localization and transcriptional factor function will be further investigated.

To identify the *PpARFs* with putative function in regulating fruit development, we analyzed their expression in S1 and S4-3 peach fruits, flower buds, flowers, young leaves, old leaves, and peach roots using real-time PCR. We found that they are expressed in all assayed peach tissues with varying expression levels, suggesting their function redundancy as well as function diversity. Nevertheless, the expression of *PpARF1*, *PpARF3, PpARF10A*, *PpARF17* and *PpARF18* significantly differed between S1 and S4, and they may activate or inhibit fruit ripening.

One important quality feature for peach fruit is its texture, being firm or soft. To figure out if any of PpARFs may be involved in regulating the fruit texture, we compared their expression in mesocarp tissue from the fruits of the melting ‘Okubo’ and stony hard ‘Jingyu’ cultivars at different developmental stages. Our results show that the expression of *PpARF4* is decreased during fruit maturation in both cultivars but its expression level strongly differs (Fig. 5a), indicating its role in peach ripening process. The expression of its tomato homolog, *SlARF4*, is higher in the pericarp tissues of immature fruit and dramatically declines at the onset of ripening, when sugar content increases [27]. *SlARF4* is known to play a role in determining fruit cell wall architecture and down-regulation of *ARF4* in tomato results in harder fruit than in the wild type [31]. Thus, *PpARF4* very likely plays the similar role in peach fruit ripening by regulating the sugar metabolism and cell wall which leads to the different fruit firmness of the two cultivars. On the contrary, the expression of *PpARF7* was increased during fruit maturation and its expression level was higher in ‘Jingyu’ (Fig. 5b). Its tomato homolog, *SlARF7*, is an auxin response–attenuating gene and acts as a negative regulator of the auxin signaling pathway [42]. Studies have shown that the softening of peach fruit is correlated to the increased auxin concentration in late fruit development [43]. The concentration of auxin is lower in stony hard fruit than in melting fruit [44]. Thus, high expression of *PpARF7* may be the response to auxin increase during fruit ripening of melting peach.

In addition to fruit development, other *PpARF*s were differentially expressed in other tissues such as *PpPARF2A*. It was expressed at the higher level in blooms than in buds. Studies in *Arabidopsis* and tomato have shown that *ARF2* is a pleiotropic developmental regulator in flower development. The silenced *AtARF2,3,4* line leads to abnormal morphology of pollen grains [45] and SlARF2 regulates flower organ senescence in tomato [46]. Thus, PpPARF2A may play the similar roles in peach. Our work also showed the higher expression of *PpARF16* and *PpARF17* in young leaves than in mature leaves. These two genes are in the same clade of the phylogenetic tree (Fig. 1), which also includes SlARF10. In tomato, down-regulation of *ARF10* leads to narrower leaflet blades with larger stomata but lower densities and water loss than wild type, suggesting that ARF10 has a function in maintaining water balance in leaves [47]. Thus, we expect that PpARF16 and PpARF17 may participate in regulating leaf water balance.

Interestingly, *PpARF2B*, *PpARF10A*, and *PpARF12* may play important roles in root development, because their expression levels in roots are significantly higher than in other tissues. In *Arabidopsis*, AtARF10 and AtARF16 inhibit differentiation of distal stem cells in roots by inhibiting transcription of the homeodomain transcription factor *WOX5* [48]. In addition, knocking out *OsARF12* in rice resulted in shortened root length, indicating that OsARF12 plays a positive role in promoting root elongation [49].

The common feature of *ARF*s is their transcriptional response to auxin. *ARF*s in maize, rice, and sweet orange respond positively to exogenous auxin but display distinct expression patterns [11, 15, 16]. The exogenous auxin analog NAA and auxin receptor inhibitor PCIB have previously been used as peach fruit treatments for purposes of studying gene function [5]. In this work, we used NAA and PCIB concentrations and treatment times established in our previous study [39]. In ‘Jingyu’ mesocarp, we observed a total of 6 *PpARF*s being upregulated to varying degrees after NAA treatment. These genes may be important for auxin-dependent transcription and post-transcriptional regulation. The expression of some genes (*PpARF2B*/*4*/*7*/*10A*) increased rapidly after 1.5 h of NAA treatment (Fig. 7), known as auxin rapid response genes. Expression of these genes then decreased rapidly and increased again under the long-term effects of NAA, which may be a protective mechanism for auxin rapid response genes [50].

In conclusion, the present study analyzed the gene and functional domain structure of *PpARF*s and their expression pattern in different tissues and fruits at different developmental stages of two peach cultivars. We identified the candidate *PpARF*s that are correlated with fruit development and involved in regulating fruit development and firmness. Our results provide the basic expression pattern of entire peach ARF family, paving the ground for studying their functions in development of diverse plant organs. Particularly understanding the functional differentiation of *PpARF* family members in peach and their regulation in fruit development and quality control will directly benefit future fruit tree breeding and engineering.

## Electronic supplementary material

Below is the link to the electronic supplementary material.

Supplementary file1 (DOCX 432 kb)
