# Supplementary material for: Genome-wide identification of the ARF (auxin response factor) gene family in peach and their expression analysis
Source: Mol Biol Rep. 2020 May 19;47(6):4331–44. doi: 10.1007/s11033-020-05525-0 (PMC7295738; doi:10.1007/s11033-020-05525-0)
Supplement: Supplementary file 1 — Supplementary file1 (DOCX 432 kb) [file 11033_2020_5525_MOESM1_ESM.docx]

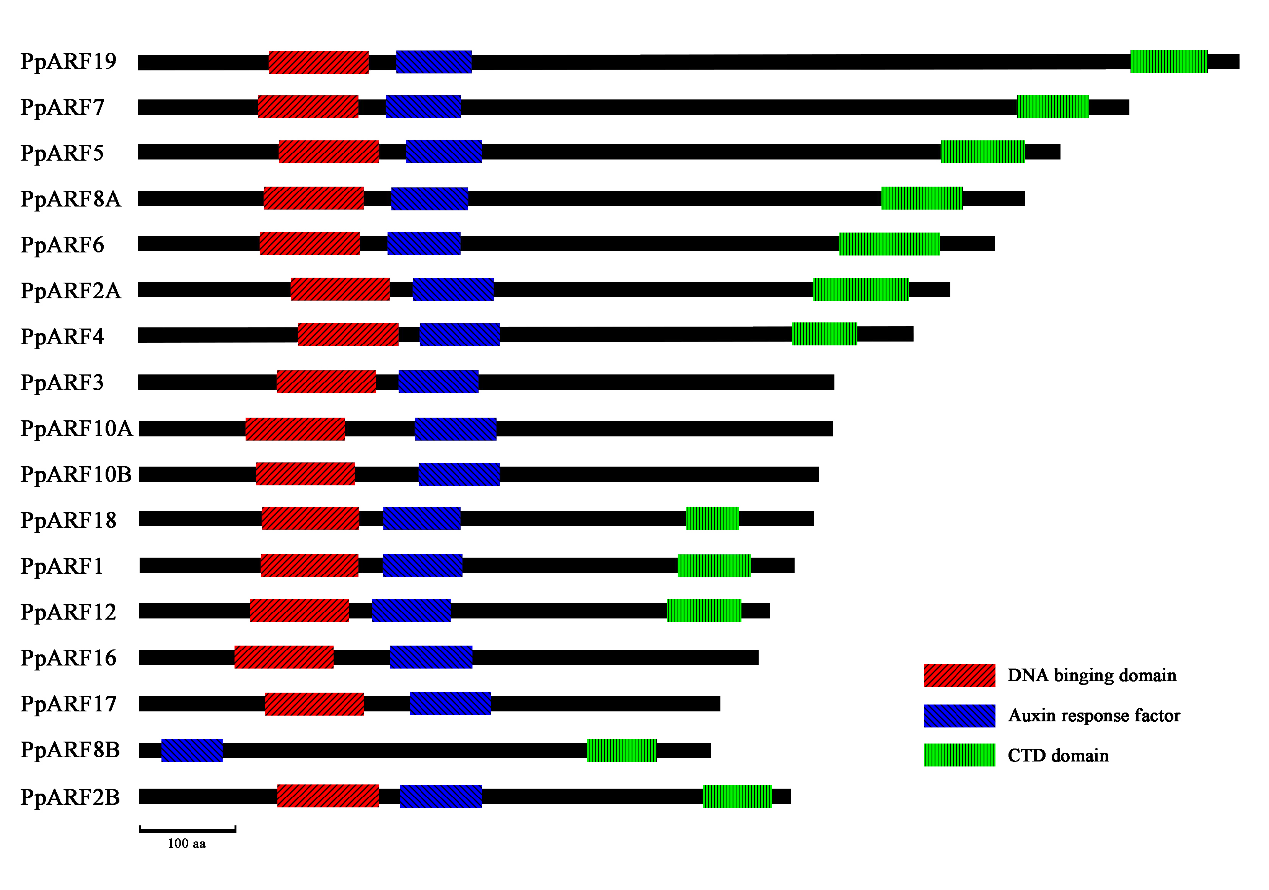
**Fig. supplementary 1** Analysis of conserved domains in PpARFs. Amino acid sequences of predicted PpARFs were aligned using the Clustal X2 program. The conserved domains were identified using NCBI web. The DNA-binding domain (red), auxin response domain (blue), and CTD (Aux/IAA family domain; green) are shown.


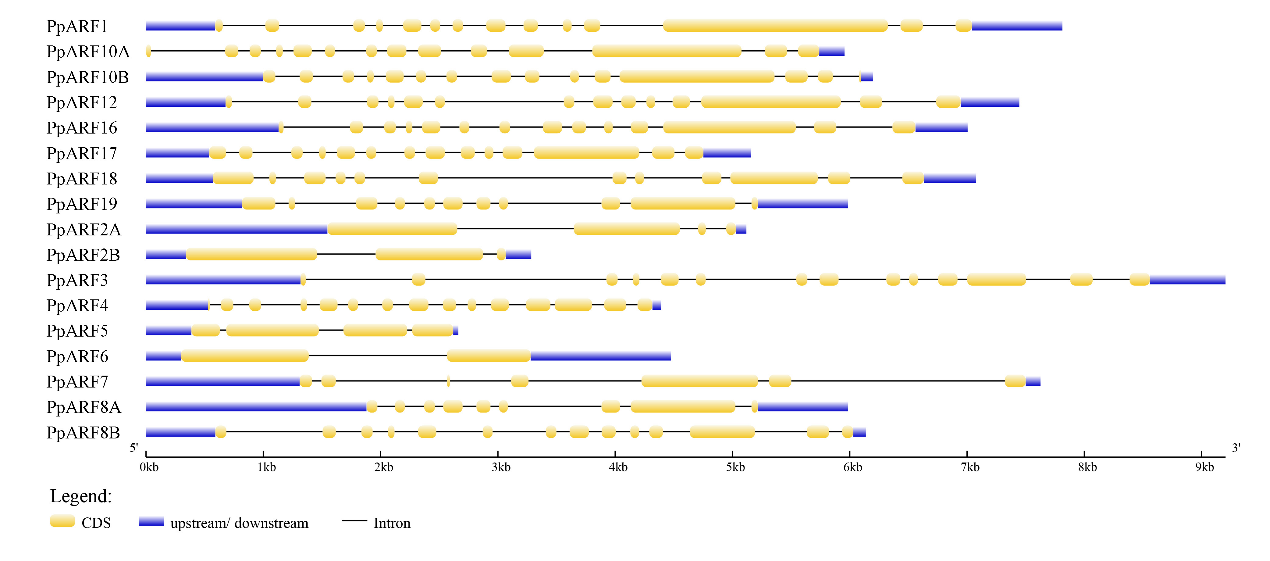
**Fig. supplementary 2** Exon-intron organization of PpARFs. The exon and introns are represented by yellow boxes and black lines, respectively. The genes structure were identified using GSDS 2.0 web
